# RUVBL1-modulated chromatin remodeling alters the transcriptional activity of oncogenic CTNNB1 in uveal melanoma

**DOI:** 10.1038/s41420-023-01429-7

**Published:** 2023-04-19

**Authors:** Chao Zhang, Shuai Wu

**Affiliations:** 1 Department of Strabismus and Pediatric Ophthalmology, the Second Hospital of Jilin University, 130041 Changchun, P. R. China; 2 Department of Orbital Disease and Ocular Plastic Surgery, the Second Hospital of Jilin University, 130041 Changchun, P. R. China

**Keywords:** Melanoma, Melanoma

## Abstract

Recent years have witnessed an increasing research interest in the therapeutic value of aberrant chromatin regulatory processes in carcinogenesis. Our study was performed to explore the possible carcinogenic mechanism of the chromatin regulator RuvB-like protein 1 (RUVBL1) in uveal melanoma (UVM). The expression pattern of RUVBL1 was retrieved in bioinformatics data. The correlation between RUVBL1 expression and the prognosis of patients with UVM was analyzed in publicly available database. The downstream target genes of RUVBL1 were predicted and further verified by co-immunoprecipitation. The bioinformatics analysis results showed that RUVBL1 may be associated with the transcriptional activity of CTNNB1 by regulating chromatin remodeling, and that RUVBL1 functioned as an independent prognostic factor for patients with UVM. The UVM cells manipulated with RUVBL1 knockdown were introduced for in vitro investigation. CCK-8 assay, flow cytometry, scratch assay, Transwell assay and Western blot analysis were used for detection on the resultant UVM cell proliferation, apoptosis, migration, invasion and cell cycle distribution. In vitro cell experimental data showed that RUVBL1 expression was significantly increased in UVM cells and RUVBL1 knockdown inhibited the proliferation, invasion and migration of UVM cells, accompanied by augmented apoptosis rate and blocked cell cycle progression. To sum up, RUVBL1 enhances the malignant biological characteristics of UVM cells by increasing the chromatin remodeling and subsequent transcription activity of CTNNB1.

## Introduction

Uveal melanoma (UVM) refers to the melanomas detected on the choroid, ciliary body, and iris of the eye and patients suffering from UVM usually undergo painless loss or distortion of vision [1]. As the most frequently occurring primary intraocular malignancy arising from melanocytes in the eye in adults, UVM is featured with specific chromosome changes and gene mutations [2, 3]. Currently, available first-line therapeutic strategies for this malignancy include resection, enucleation and radiation therapy yet long-term survival rate remains satisfactory [4]. The complex interaction among genetic, molecular signaling, and prognostic profiles has been attracting research attention to develop promising treatment modalities [5]. However, the molecular mechanism implicated in UVM has not been fully answered.

Of note, chromatin remodeling has been documented to determine diverse processes, including gene transcription and cell fate, and is implicated in the occurrence and development of various tumors [6]. In recent years, targeting chromatin remodeling via related regulatory factors based on small molecules has emerged as a potential therapeutic intervention in the context of oncology [7, 8]. For instance, a transcriptional program involving SWI/SNF chromatin remodeling complex has been reported as a novel path against UVM through mediation on malignant cell survival [9]. RuvB-like protein 1 (RUVBL1) and RUVBL2 are known as two highly conserved AAA + ATPases and can form a hetero-hexameric complex implicated in multiple cellular events, with chromatin remodeling included [10].

In addition, RUVBL1 has been demonstrated to affect the progression of osteosarcoma by regulating chromatin remodeling and transcriptional activity of β-catenin/lymphoid enhancer-binding factor 1 complex [11]. Given the aforementioned evidence and bioinformatics analysis prior to our investigation, RUVBL1 was identified as a potential regulator in chromatin remodeling of UVM, probably depending on transcriptional activity of β-catenin1 (CTNNB1). CTNNB1 has been highlighted to harbor tumor suppressive property functioning as a hub gene and core biomarker in UVM [12]. More importantly, upregulated CTNNB1 has been documented as an oncogene signature in triple-negative breast cancer accompanied by the inactivation of SMARCA4, an ATPase subunit of the SWI/SNF chromatin remodeling complex [13], suggesting the potential implication of CTNNB1 in chromatin remodeling in tumorigenesis.

Considering the existing evidence and reports, we attempted to probe into the underlying molecular mechanism by which RUVBL1 may play a role in mediating UVM progression through regulating chromatin modeling and CTNNB1.

## Results

### RUVBL1 is associated with chromatin remodeling in UVM cells

**Fig. 1:**
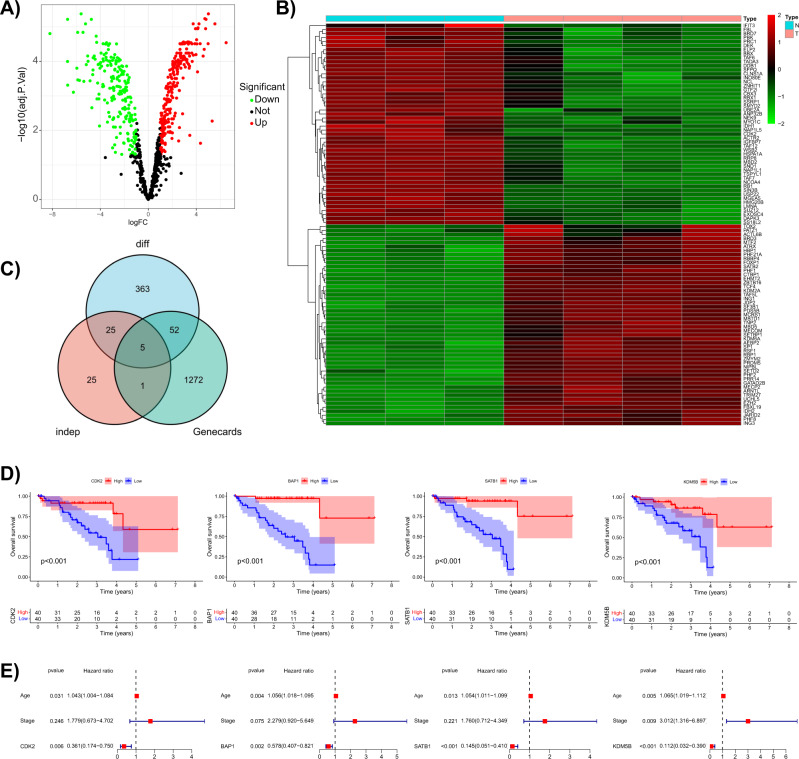
Bioinformatics analysis of target genes implicated in chromatin remodeling in UVM. **A** Volcano plot on differential analysis of 812 chromatin regulators in the microarray dataset GSE62075 from GEO database (each point represents a gene, red indicates the significantly highly expressed genes, green indicates the significantly poorly expressed genes, and black indicates non-differentially expressed genes). **B** Heatmap of the difference analysis showing the expression of the 50 most significantly upregulated and downregulated genes, each column represents a single sample, each row represents a single gene, and the color means the same with panel (**A**). **C** Venn diagram of the intersection of the DEGs, independent prognostic factors, and UVM-related genes, revealing 5 candidate genes. **D** Survival analysis regarding the expression of CDK2, BAP1, SATB1, and KDM5B (the top half is a survival curve diagram, the abscissa is the survival time, the ordinate is the survival rate, red and blue curves indicate the high and low expression, respectively; *p* < 0.001 indicates a significant difference with regard to survival time between the two groups; the lower half indicates the number of remaining patients in the two groups at each survival time point. **E** The multivariate-independent prognostic analysis of CDK2, BAP1, SATB1, and KDM5B (each row represents one factor, *p* < 0.05 indicates that the factor is associated with survival, HR > 1 means that the point is on the right of the dotted line and represents a high risk factor, HR < 1 means that the point is on the left side of the dotted line and represents a low-risk factor).

To identify the genes that is of importance to chromatin remodeling in UVM cells, multiple databases were jointly analyzed and downstream pathways were searched. Microarray data including normal samples and tumor samples were retrieved from the GEO database, and dataset GSE62075 was selected for subsequent analysis. R software was used to obtain expression data of 812 chromatin regulators [14]. Given that some samples in the dataset were transfected, 4 tumor cell samples and 3 UVM cell samples were selected for gene differential expression analysis, and 445 DEGs were obtained (Fig. 1A, B). There were 238 up-regulated genes, which were labeled in red in the volcano plot and heatmap, mainly including ING1, ZBTB16, CTBP1, EHMT2, and NIPBL. There were 207 down regulated genes, which were labeled in green in the volcano plot and heatmap, mainly including RB1, SIN3B, WSB2, USP22, and HSPA1A.

Meanwhile, the expression data and clinical information of 80 patients with UVM were downloaded from TCGA database, and the expression of 854 chromatin regulatory factors in 80 samples were screened out for survival analysis. In total, 129 genes related to survival condition of patients with UVM were identified and further analyzed, 56 of which were finally obtained as independent prognostic factors.

In addition, a total of 1330 UVM-related genes were retrieved from GeneCards database. As revealed from the intersection (Fig. 1C), 5 candidate genes (CDK2, BAP1, SATB1, KDM5B, and RUVBL1) were attained, 4 of which (CDK2, BAB1, SATB1, and KDM5B) were further subjected to survival analysis and independent prognosis analysis (Fig. 1D, E). The results suggested that patients with high expression of CDK2, BAP1, SATB1, and KDM5B had significantly longer survival time than those with low expression. Independent prognostic analysis showed that the gene expression of CDK2, BAP1, SATB1, and KDM5B could predict the risk of UVM as independent risk factors. Moreover, the HR values of these four genes were less than 1, indicating that the individuals with high expression of CDK2, BAP1, SATB1, and KDM5B had low risk of UVM. The contribution of RUVBL1 to tumor progression has been reported to rely on chromatin remodeling [11]. Therefore, RUVBL1 was selected as the target gene for subsequent investigation.

### RUVBL1 serves as an independent prognostic factor in UVM

**Fig. 2:**
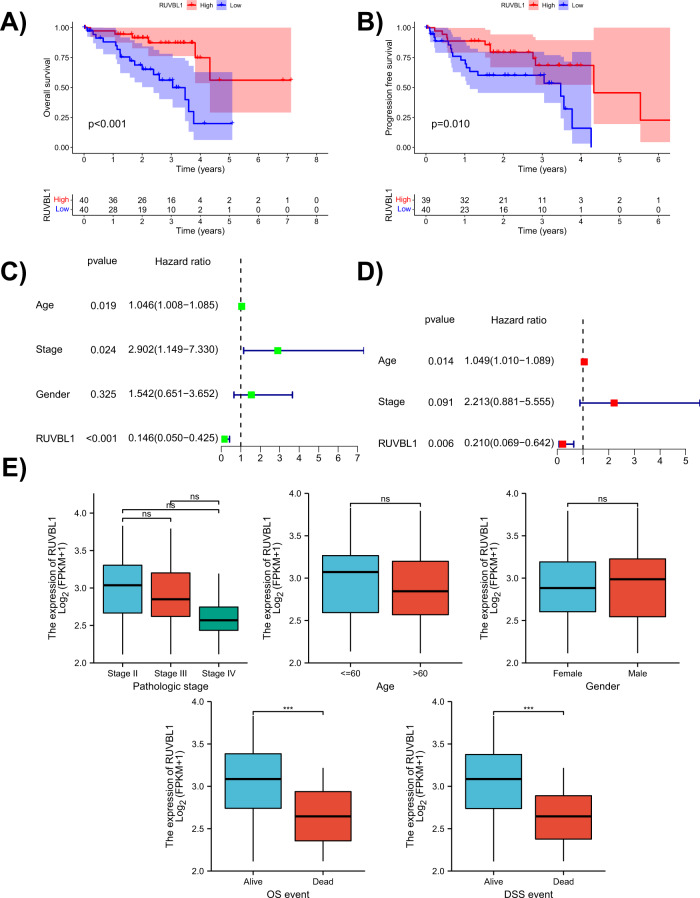
Correlation between RUVBL1 and prognosis of patients with UVM based on the TCGA database. **A** Survival analysis of patients with high and low RUVBL1 expression in the TCGA database (the top half is a survival curve diagram, the abscissa is the survival time, the ordinate is the survival rate, red and blue curves indicate the patients with high and low expression of RUVBL1, respectively, *p* < 0.001 indicates a significant difference regarding survival time between the two groups; the lower half indicates the number of remaining patients in the two groups at each survival time point. **B** Progression-free survival analysis of the patients with high and low RUVBL1 expression in the TCGA database (the top half is a progression-free survival curve, the abscissa is the survival time, the ordinate is the progression-free survival, red and blue curves indicate the patients with high and low expression of RUVBL1, respectively, *p* < 0.05 indicates that RUVBL1 is associated with progression-free survival; the lower half indicates the number of remaining patients in the two groups at each survival time point. **C** Forest plot of univariate independent prognosis analysis f RUVBL1 and age, stage or gender. **D** Forest plot of multivariate independent prognostic analysis of RUVBL1 and age or stage (each row represents one factor, *p* < 0.05 indicates that the factor is associated with survival, HR > 1 means that the point is on the right of the dotted line and represents a high risk factor, HR < 1 means that the point is on the left side of the dotted line and represents a low risk factor; *p* < 0.05 indicates that the factor serves as an independent prognostic factor). **E** The correlation analysis of RUVBL1 and each clinical characteristic (the abscissa represents the grouping of the clinical characteristics, the ordinate indicates the expression of RUVBL1, ns indicates no statistical difference between the two groups, ***p < 0.001).

To validate the clinical relevance of RUVBL1, we extracted RUVBL1 expression in 80 samples from TCGA database and conducted survival analysis (Fig. 2A), progression-free survival analysis (Fig. 2B) and clinical correlation analysis combined with the clinical information. The results showed that both the overall survival rate and the progression-free survival rate varied significantly between the patients with UVM carrying high and low RUVBL1 expression. Subsequently, age, gender and tumor stage were selected from clinical information for univariate and multivariate independent prognostic analysis (Fig. 2C, D). RUVBL1 was revealed as an independent prognostic factor for patients with UVM. Finally, the correlation analysis of RUVBL1 with various clinical features indicated that RUVBL1 was significantly associated with overall survival and disease-specific survival (Fig. 2E).

### RUVBL1 enhances transcriptional activity of CTNNB1

**Fig. 3:**
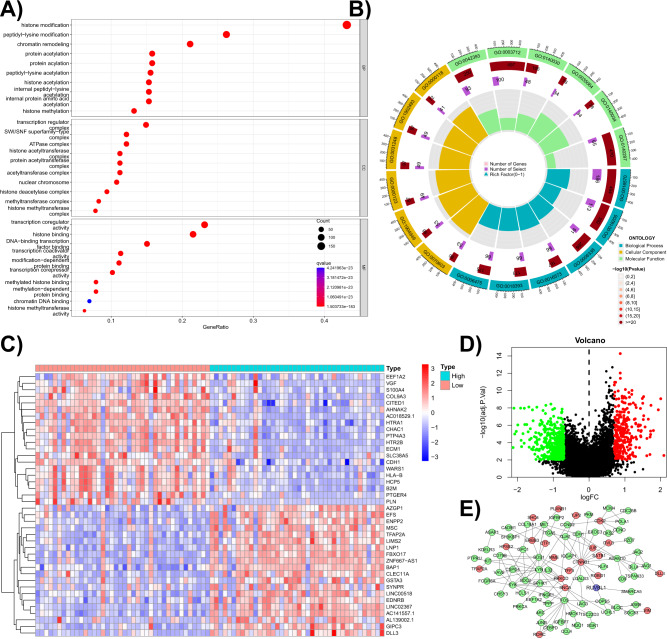
Online database analysis for the biological function of RUVBL1. **A** The bubble chart displaying GO enrichment analysis of the DEGs in the GEO database (the abscissa indicates the proportion of the genes, the ordinate indicates the GO node, each circle represents the DEGs enriched at the node, larger circles reflect more enriched genes, and the deeper color of the circle reflects more significant enrichment of the gene at the node). **B** The circle chart displaying GO enrichment analysis of DEGs in the GEO database; the outermost circle is the biological process (blue), molecular function (green) and cellular composition (yellow) of the GO analysis and the number is the GO node number; the second circle is the number of genes enriched to each GO node and the deeper color reflects more significant enrichment of the DEGs; the third circle is the number of DEGs at each GO node; the innermost circle represents the gene ratio). **C** Heatmap of differential analysis of TCGA database showing the expression level of the 20 most significantly upregulated (red) and downregulated (blue) genes in the high and low RUVBL1 expression groups; the darker the color, the more significant the difference. **D** The volcano plot of differential analysis on TCGA database (each point represents the expression of a gene, red indicates significantly high expression, and green indicates significantly low expression). **E** PPI network of DEGs in TCGA (each circle represents a gene, upregulated genes are shown in red, downregulated genes in green, and the target gene, RUVBL1, in blue).

To explore the functional role of RUVBL1 and the downstream genes, the GO enrichment analysis of the DEGs in the microarray dataset from GEO was performed (Fig. 3A, B). The analysis results of biological process identified RUVBL1 to be mainly involved in the biological processes related to chromatin remodeling, such as histone modification, DNA repair regulation, DNA reorganization, DNA conformational change and double-stranded unfolding, in tumor cells.

Further investigation was performed for the downstream pathway of RUVBL1. TCGA data were divided into high and low-expression groups according to the expression of RUVBL1 for differential expression analysis, revealing a total of 283 upregulated genes (labeled in red in the volcano plot) and 466 downregulated genes (labeled in green in the volcano plot). Moreover, we performed heatmap analysis of the top-ranked differentially expressed genes, genes significantly upregulated in the RUVBL1 high expression group mainly included EEF1A2, VGF, S100A4, etc., while genes significantly upregulated in the RUVBL1 low expression group mainly included AZGP 1, EFS, ENPP 2, etc (Fig. 3C, D). PPI analysis on DEGs from TCGA database using the STRING database validated CTNNB1, SMARCA5 and COPS5 as three proteins associated with RUVBL1-related pathways (Fig. 3E).

**Fig. 4:**
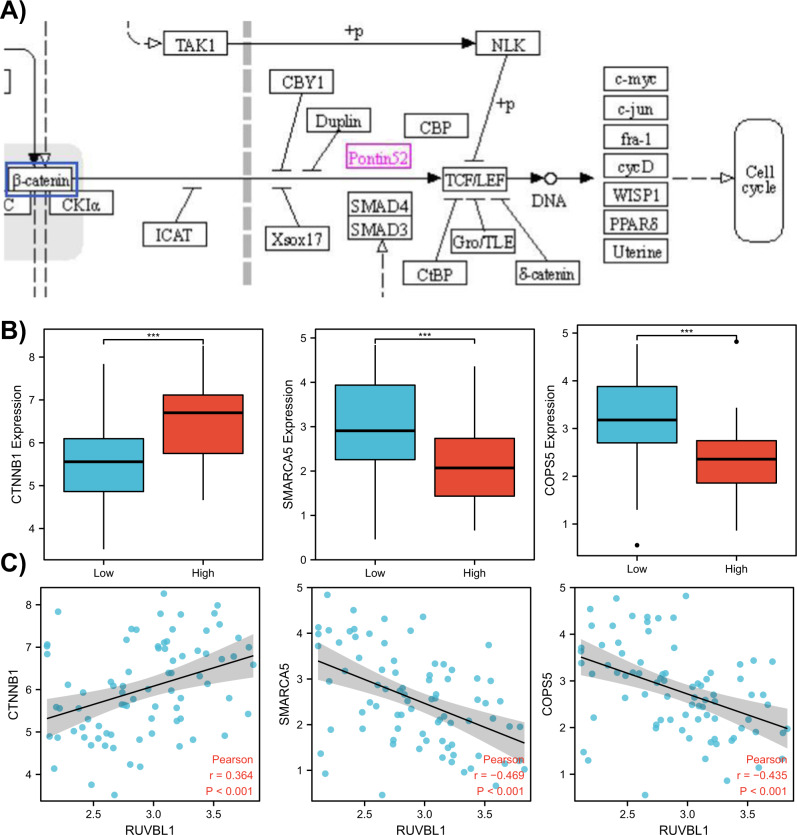
Correlation analysis between RUVBL1 and CTNNB1. **A** RUVBL1-related signaling pathway diagram of KEGG database (Pontin53 is the target gene RUVBL1, and the blue box is the downstream gene CTNNB1). **B** Expression of CTNNB1, SMARCA5, and COPS5 in the high and low RUVBL1 expression groups (the red column shows the expression level in the high expression group, and the blue column shows the expression level in the low expression group). **C** Pearson correlation analysis between CTNNB1, SMARCA5, or COPS5 and RUVBL1, respectively (each point is the expression level of RUVBL1 and one related gene in the same sample, *p* < 0.001 indicates a significant correlation).

To determine the downstream genes of RUVBL1, the signaling pathway map of RUVBL1 was retrieved from the KEGG database (Fig. 4A). Based on the PPI network, RUVBL1 enhanced the transcriptional activity of CTNNB1. For verification purpose, we also compared the expression levels of CTNNB1, SMARCA5, and COPS5 between the high and low RUVBL1 expression groups in the TCGA database (Fig. 4B), and conducted the Pearson Correlation Coefficient analysis on RUVBL1 (Fig. 4C). It was found that the expression level of CTNNB1 was significantly increased in the high RUVBL1 expression group, along with a significantly positive correlation between RUVBL1 and CTNNB1 expression level.

The above results evidenced that RUVBL1 enhanced the transcriptional activity of CTNNB1 by promoting chromatin remodeling.

### Knockdown of RUVBL1 inhibits the malignant biological features of UVM cells

**Fig. 5:**
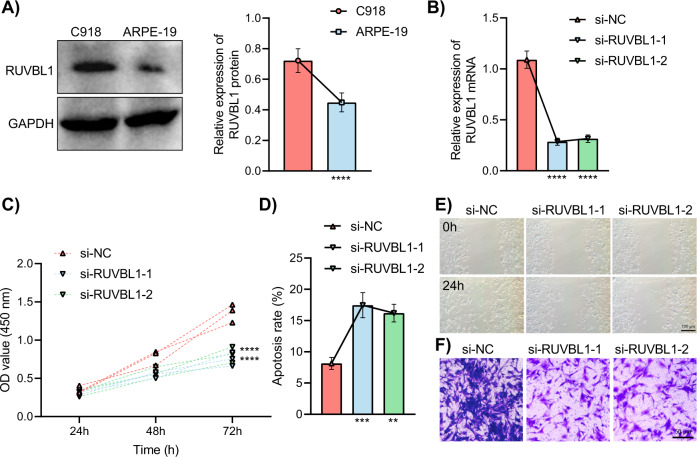
Effects of RUVBL1 knockdown on the biological properties of UVM cells. **A** The expression of RUVBL1 in the C918 and ARPE-19 cells determined by Western blot analysis, ****, *p* < 0.0001. **B** The relative expression level of RUVBL1 in C918 cells transfected with si-RUVBL1-1 and si-RUVBL1-2 determined by RT-qPCR, ****p < 0.0001. **C** The proliferative capacity of C918 cells transfected with si-RUVBL1-1 and si-RUVBL1-2 determined by CCK-8 assay; OD values represent the cell viability; higher OD value reflects stronger cell proliferation ability, ****p < 0.0001. **D** The apoptosis rate of C918 cells transfected with si-RUVBL1-1 and si-RUVBL1-2 measured by flow cytometry, **p < 0.01, ***p < 0.001. **E** The migrative ability of C918 cells transfected with si-RUVBL1-1 and si-RUVBL1-2 detected by scratch assay; the portion of the black shaded area in the middle of the si-RUVBL1-1/2 groups was significantly larger, highly suggestive of reduced migration ability. **F** The invasive capacity of C918 cells detected by Transwell assay; the counting suggested that RUVBL1 knockdown significantly reduced C918 cell invasion capacity.

To verify the above bioinformatics analysis, Western blot analysis was carried out to quantify the RUVBL1 expression in human UVM cell line C918 and human retinal pigment epithelial cell line ARPE-19 (Fig. 5A). The expression of RUVBL1 in C918 cells was found to be significantly higher when compared with ARPE-19 cells.

For further exploration regrading the effect of RUVBL1 on the biological characteristics of UVM cells, C918 cell line was transfected with si-NC, si-RUVBL1-1, and si-RUVBL1-2. RT-qPCR results showed that RUVBL1 expression in C918 cells transfected with si-RUVBL1-1 and si-RUVBL1-2 was significantly lower than that those transfected with si-NC (Fig. 5B).

Cell proliferation was further measured by CCK-8 assay (Fig. 5C), apoptosis by flow cytometry (Fig. 5D), cell migration by scratch assay (Fig. 5E) and invasion ability Transwell assay (Fig. 5F). The survival rate was significantly diminished and apoptosis was promoted by transfection of si-RUVBL1-1 and si-RUVBL1-2 along with reduced scratch healing rate and smaller number of invasive C918 cells.

The above results demonstrated that malignant characteristics of UVM cells were suppressed more significantly by si-RUVBL1-1 and si-RUVBL1-2 when compared with si-NC.

### Knockdown of RUVBL1 inhibits CTNNB1 transcriptional activity, thereby blocking the UVM cell cycle progression

**Fig. 6:**
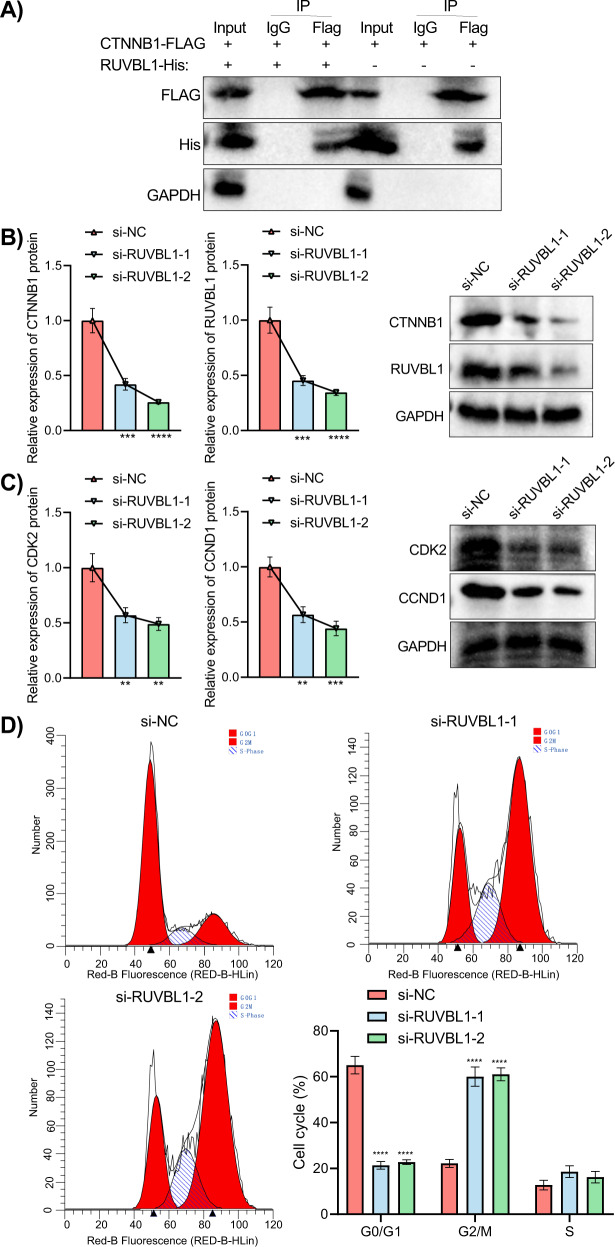
Effect of RUVBL1 knockdown-mediated CTNNB1 transcriptional activity on the UVM cell cycle. **A** Western blot analysis on the co-IP assay of the relationship between RUVBL1 and CTNNB1. RUVBL1, or CTNNB1 was immunoprecipitated with anti-his or anti-Flag and immunoblotted for the indicated proteins. **B** RUVBL1 or CTNNB1 expression determined by Western blot analysis after transfection with si-RUVBL1-1 and si-RUVBL1-2. **p < 0.01, ***p < 0.001. **C** CDK2 or CCND1 protein levels determined by Western blot analysis after transfection with si-RUVBL1-1 and si-RUVBL1-2. **p < 0.01, ***p < 0.001. **D** Flow cytometric detection of cell cycle distribution after transfection with si-RUVBL1-1 and si-RUVBL1-2. ****p < 0.0001. The comparison of the data between the above groups was performed by ANOVA.

The study focus was then shifted to whether the functional role of RUVBL1 in UVM progression was achieved by regulating CTNNB1 expression. According to co-IP assay (Fig. 6A), RUVBL1 targeted CTNNB1.

Furthermore, the resultant expression of RUVBL1 and CTNNB1 in transfected cells was determined by Western blot analysis (Fig. 6B). The results revealed that, as compared with si-NC, the expression of CTNNB1 and RUVBL1 was reduced in response to si-RUVBL1-1 or si-RUVBL1-2. To further validate the effect of highly expressed CTNNB1 on the cell cycle, the expression of the cell cycle-related regulators CDK2 and CCND1 was examined by Western blot analysis (Fig. 6C), results of which revealed significantly lower levels of CDK2 and CCND1 in presence of si-RUVBL1-1 or si-RUVBL1-2. We further analyzed the cell cycle distribution in cells in response to si-RUVBL1-1 or si-RUVBL1-2 by flow cytometry. It was noted that cells in the G2 phase were significantly increased in the presence of si-RUVBL1-1 or si-RUVBL1-2, indicating that knockdown of RUVBL1 could arrest UVM in G2 phase (Fig. 6D).

The above results signaled that RUVBL1 promoted mitosis in UVM cells by activating CTNNB1, and that knockdown of RUVBL1 inhibited CTNNB1 transcriptional activity to arrest the UVM cell cycle.

## Discussion

**Fig. 7:**
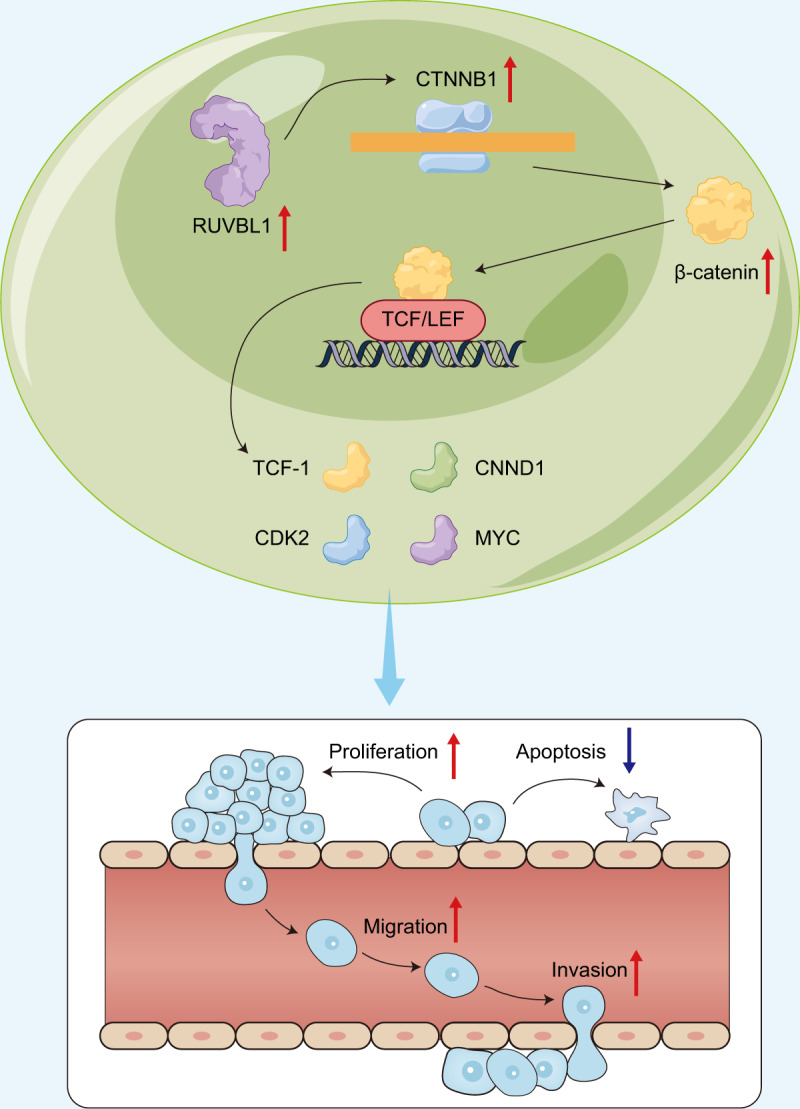
Molecular mechanism graph of RUVBL1 in UVM, where RUVBL1 promotes the transcriptional activity of CTNNB1 through chromatin remodeling, leading to augmented malignant phenotypes of UVM.

UVM is regarded as an intraocular malignancy characterized by a risk of liver metastasis while better understanding on genetic and molecular factors may warrant favorable prognosis [15]. Notably, the contribution of chromatin regulatory procedures to carcinogenesis has been recently acknowledged [16]. Based on the bioinformatics analysis and in vitro cell experiments, it was reported in the present study that RUVBL1 may aggravate the malignant biological characteristics of UVM cells by promoting chromatin remodeling, and enhancing the transcription activity of CTNNB1, thus in contribution to the progression of UVM (Fig. 7).

The initial finding from this study supported that RUVBL1 was expressed at a high level in UVM samples and cells, acting as an independent prognostic factor. Largely in agreement with our finding, highly expressed RUVBL1 in non-small cell lung cancer has been reported to be indicative of undesirable oncogenic outcomes of patients, serving as a promising prognostic biomarker [17]. Also, the prognostic and diagnostic potential of RUVBL1 in oral squamous cell carcinoma has been uncovered [18]. Furthermore, the implication of RUVBL1 in differentiation, proliferation and invasion of head and neck squamous cell carcinoma cells has been documented by incorporating histone variant H2AZ in chromatin, highly suggestive of its functional role in evaluating prognosis [19]. In addition, silence of RUVBL1 exerts inhibitory effect on malignant cell proliferation both in vitro and in vivo in the context of prostate tumor as well as lung adenocarcinoma [20, 21]. Further exploration through loss-of-function assays in our study unmasked the anti-tumor property of RUVBL1 depletion via siRNA as evidenced by suppressed proliferation, migration and invasion of UVM cells as well as promoted cell apoptosis while blocking cell cycle. RUVBL1 has been deciphered to mediate mitosis and proliferation that knockdown of RUVBL1 triggers defects in chromosome segregation and alignment [22]. Consistently, dysregulated RUVBL1 has been signaled to trigger replication-transcription interference and impairs genome integrity during S-phase [23]. Likewise, a mechanistic report has pointed to the anti-proliferative activity of downregulated RUVBL1 in lung adenocarcinoma by arresting G1/S phase cell cycle [24], supporting the validation of our experimental data.

More importantly, the subsequent mechanistic investigation unveiled that blockade on UVM cell cycle progression elicited by silenced RUVBL1 depended on inhibited transcription activity of CTNNB1 accompanied by diminished expression levels of CDK2 and CCND1. According to observations and evaluations made in the study of Wen et al., the promoted transcription activity of CTNNB1 has been detected simultaneously with strengthened migrative and invasive capabilities of colon cancer cells [25]. The inhibited transcription activity of CTNNB1 has also been observed to be coincident with restrained progression of gastric cancer and the inactivation of the Wnt/β-catenin signaling pathway [26]. Similarly, protein levels of both β-catenin and CDK2 have been elevated in melanocytes stimulated with melanogenesis [27]. Evidence has been presented demonstrating that CCND1 participates in the tumor-suppressive action of the E3 ligase adaptor, autophagy and beclin 1 regulator 1 to limit the growth of UVM cells [28]. The significance of β-catenin-dependent transcription activity to oncogenic output modulated by chromatin regulators has been accentuated in colorectal cancer as well [29]. This study used different methods to explain their interaction, and found that RUVBL1 enhanced the transcriptional activity of CTNNB1 by promoting chromatin remodeling. Besides, RUVBL1 also regulated CTNNB1 by direct protein binding. However, this study fails to clarify the proportion of the two modes and whether the overlapping occurs between the two regulatory modes. Future studies are needed to verify the specific binding site between RUVBL1 and CTNNB1, and then explore the two regulatory mechanisms deeply by constructing mutant proteins.

Collectively, our findings on the tumor-supporting action of RUVBL1 provide new insights into the mechanism of UVM and offer potential targets for chromatin remodeling. The present study revealed the molecular mechanism by which RUVBL1 affects the progression of UVM and laid a theoretical basis for clinical treatment and prognostic assessment. However, due to the paucity of in vivo assays, the same should also be applied to results generated from human UVM tissues and animal models to validate our in vitro findings, as well as elucidate novel therapeutic targets for clinical application in the future.

## Materials and methods

### Bioinformatics analysis

Through the GEO database, the UVM-related gene expression microarray dataset was downloaded, and the chromatin regulators were selected [14]. Differential analysis was performed by the limma package of the R software to screen out differentially expressed genes (DEGs) between the UVM cells, and normal uveal melanocytes. The gene expression matrices and clinicopathological data for patients with UVM were downloaded from the TCGA database, and the chromatin regulators were filtered using survival package of R software regarding the potential of being survival and independent prognostic factors The datasets of genes as independent prognostic factors for patients with UVM were attained. Next, the UVM-related genes were obtained through the GeneCards database. RUVBL1 was identified as the target gene by taking the intersection of the DEGs, the independent prognostic genes, and the UVM-related genes.

The UVM patient samples from the TCGA database were assigned into two groups regarding the expression pattern of RUVBL1 as the RUVBL1 high expression group and RUVBL1 low expression group, the DEGs associated with RUVBL1 were selected by differential analysis, and the protein-protein interaction (PPI) network was drawn using the STRING database. The signaling pathways involving RUVBL1 was retrieved using the KEGG database and CTNNB1 was determined as the downstream regulatory gene of RUVBL1. The correlation between CTNNB1 and RUVBL1 was analyzed by the Pearson correlation coefficient based on the clinicopathological data of patients with UVM from the TCGA database.

### In vitro cell culture and protocols

The human UVM cell line C918 and the human retinal pigment epithelial cell line ARPE-19 were both purchased from Biobw (Beijing, China). Cells were cultured in RPMI-1640 medium (Thermo Fisher Scientific, Waltham, MA) containing 10% fetal bovine serum and 10% penicillin-streptomycin in an incubator at 37 °C with 5% CO_2_.

Cells were seeded in 6-well plates (3 × 10^5^ cells/well), cultured for 24 h for transfection and then transfected according to Lipofectamin 2000 instructions (Invitrogen, New York, CA) with liposomes of scramble RNA sequence as the negative control (NC), small interfering RNA-1 (siRNA-1) against RUVBL1 (si-RUVBL1-1), and si-RUVBL1-2. Culture continued for another 24 h after transfection for subsequent experiments. siRNA sequence design was completed through the Thermo Fisher online primer design website and biosynthesized by Shanghai GenePharm (Shanghai, China), with sequence information shown in Table S1.

### Reverse transcription quantitative polymerase chain reaction (RT-qPCR)

Total RNA of the cells was extracted using Trizol (Invitrogen) and reversely transcribed into cDNA using a RT kit (RR047A, Takara, Japan) in real-time fluorescence qPCR instrument (ABI7500, Applied Biosystems, Foster City, CA). RT-qPCR was performed according to the instructions of TaqMan Gene Expression Assays protocol (Applied Biosystems). Primers as shown in Table S2 were designed on NCBI. As normalized to glyceraldehyde-3-phosphate dehydrogenase (GAPDH), 2^-ΔΔCt^ method was used to quantify gene expression.

### Western blot analysis

The total protein was extracted from cells using RIPA lysis (Beyotime Biotechnology, Shanghai, China) containing PMSF (Thermo Fisher Scientific), and the protein concentration was determined using the BCA kit (20201ES76, Yeasen Biotechnology Co., Ltd., Shanghai, China). Quantification was performed according to different concentrations, and the protein was transferred onto a PVDF membrane (Millipore, Billerica, MA) through polyacrylamide gel electrophoresis. The membrane was blocked in 5% bovine serum albumin for 1 h at room temperature and incubated with primary antibodies against RUVBL1 (ab51500, 1: 100, Abcam, Cambridge, UK), CTNNB1 (ab273712, 1: 1000, Abcam), CDK2 (ab32147, 1: 1000, Abcam), CCND1 (ab16663, 1: 200, Abcam) at 4 °C overnight. The membrane was re-probed with horseradish peroxidase-labeled goat anti-rabbit IgG (ab6721, 1: 5000, Abcam) or goat anti-mouse IgG (ab6789, 1: 5000, Abcam) for 1 h at room temperature. The luminescent solution was added for development. Protein quantification analysis was performed by ImageJ software (National Institutes of Health) as normalized to GAPDH.

### Co-Immunoprecipitation (Co-IP) assay

The transfected cells were lysed in lysis buffer (50 mM Tris-HCl pH 7.4, 150 mM NaCl, 10% glycerol, 1 mM EDTA, 0.5% NP-40 and protease inhibitor). The cell debris was removed by centrifugation. The supernatant was collected and incubated with anti-RUVBL1 or anti-CTNNB1 (Sigma-Aldrich, St. Louis, MO) and protein A/G beads (Santa Cruz Biotechnology, Santa Cruz, CA) for 2 h. After three washes using the lysis, the beads were boiled at 100 °C for 5 min. Proteins were separated by sodium dodecyl sulfate-polyacrylamide gel electrophoresis, and transferred onto a nitrocellulose membrane (Millipore), followed by immunoblotting.

### Cell counting kit-8 (CCK-8) assay

Cell proliferation was measured using the CCK-8 kit. The cells to be tested were detached into a single cell suspension (1000 cells/μL) and every 200 L cell suspension was added per well in a 96-well plate. After 0 h, 24 h, 48 h, 72 h, 96 h and 120 h, 20 μL CCK-8 solution was added to each well, and the optical density (OD) value was determined by a microplate meter at 450 nm to plot the cell proliferation curve.

### Flow cytometry

Cells to be tested were rinsed three times with pre-cooled PBS and cell apoptosis was detected with reference to the instructions of Annexin V-FITC/PI Apoptosis Detection Kit (BD Pharmingen, San Diego, CA) as previously described [30]. Cells were resuspended in 500 μL binding buffer, and incubated with 5 μL Annexin V-FITC and 5 μL PI for 15 min under condition void of light, and apoptosis was detected by flow cytometer (BD FACSCalibur).

In terms of cell cycle distribution [31], the fixed cells were washed twice with pre-cooled PBS, and moved to a 1.5 mL EP tube. Then, cells were resuspended in 20 μL RNase (working concentration of 50 μg/mL), followed by digestion in water bath at 37 °C for 30 min. The PI (20 μL, final concentration of 50 μg/mL) was used to stain the cells void of light at 4 °C for 30 min. Cell cycle distribution was detected within 24 h, with data analysis using the Flow Jo software.

### Scratch assay

Cell migration ability was determined by scratch assay, and the cells to be tested were cultured in 6-well plates. After the cells were grown to 80% confluence, a straight line was drawn using a 20 μL sterile pipette. Wound healing was examined at 0 h and 48 h after the scratch, and the cell scratch area was calculated using the WimScratch online image analysis system. The healing rate was calculated as follows: scratch healing rate = (scratch area immediately after a scratch - scratch area 48 h after a scratch)/scratch area immediately after a scratch × 100%.

### Transwell assay

At 48 h post-transfection, the cells to be tested were washed twice with PBS after detachment and resuspended in serum-free DMEM. The chambers of the Transwell assay were precoated with matrix gel according to the manufacturer’s instructions (BD Science, Bedford, MA). Then, 200 μL cells were added to the upper chamber (5 × 10^4^ cells/well for the migration assay, 1 × 10^5^ cells/well for the invasion assay). The 300 μL DMEM containing 10% fetal bovine serum was added to the lower chamber. After 24 h, the cells were removed from the upper chamber, gently washed twice with PBS, and fixed with 4% paraformaldehyde for 30 min. Cells were then stained with 1% crystal violet solution for 10 min, and counted by capturing images with a microscope.

### Statistical analysis

SPSS21.0 (IBM Corp., Armonk, NY) was used for statistical analysis. Measurement data were represented using mean ± standard deviation. The unpaired data in normal distribution and homogeneity of variance were compared using an unpaired *t* test between two groups. Data comparison among multiple groups was performed by one-way analysis of variance (ANOVA), followed by Tukey’s post hoc test. Data comparison at different time points were analyzed using repeated measures ANOVA in combination with Bonferroni correction. The Pearson correlation coefficient was used to analyze the correlation between the two indicators. The difference was statistically significant as *p* < 0.05.

## Supplementary information

Supplementary Tables

Original Data File

## Data Availability

The datasets generated and/or analyzed during the current study are available in the manuscript and supplementary materials.
