# Supplementary material for: RUVBL1-modulated chromatin remodeling alters the transcriptional activity of oncogenic CTNNB1 in uveal melanoma
Source: Cell Death Discov. 2023 Apr 19;9:132. doi: 10.1038/s41420-023-01429-7 (PMC10115834; doi:10.1038/s41420-023-01429-7)
Supplement: Supplementary file 1 — Supplementary Tables [file 41420_2023_1429_MOESM1_ESM.docx]

**Table S1** siRNA sequences

| siRNA | Sequences (5'-3') |
| --- | --- |
| si-NC | GUAUGACAACAGCCUCAAGTT |
| si-RUVBL1-1 | GAGAGGCATGTGGCGTCATAGTAGA |
| si-RUVBL1-2 | CATGTGGCGTCATAGTAGAATTAAT |

Note: si-, siRNA, small interfering RNA; RUVBL1, RuvB-like protein.

**Table S2** Primer sequences for reverse transcription quantitative polymerase chain reaction

| Gene | Sequences (5'-3') |
| --- | --- |
| RUVBL1 | Forward: GATTGGCACCAAGACCACAC |
|  | Reverse: CAATGCTGTCCTTCCCGTTG |
| GAPDH | Forward: GCACCGTCAAGGCTGAGAAC |
|  | Reverse: TGGTGAAGACGCCAGTGGA |

Note: RUVBL1, RuvB-like protein; GAPDH, glyceraldehyde-3-phosphate dehydrogenase.
