# Supplementary material for: RUVBL1-modulated chromatin remodeling alters the transcriptional activity of oncogenic CTNNB1 in uveal melanoma
Source: Cell Death Discov. 2023 Apr 19;9:132. doi: 10.1038/s41420-023-01429-7 (PMC10115834; doi:10.1038/s41420-023-01429-7)

Figure 5A

Figure 5A-1

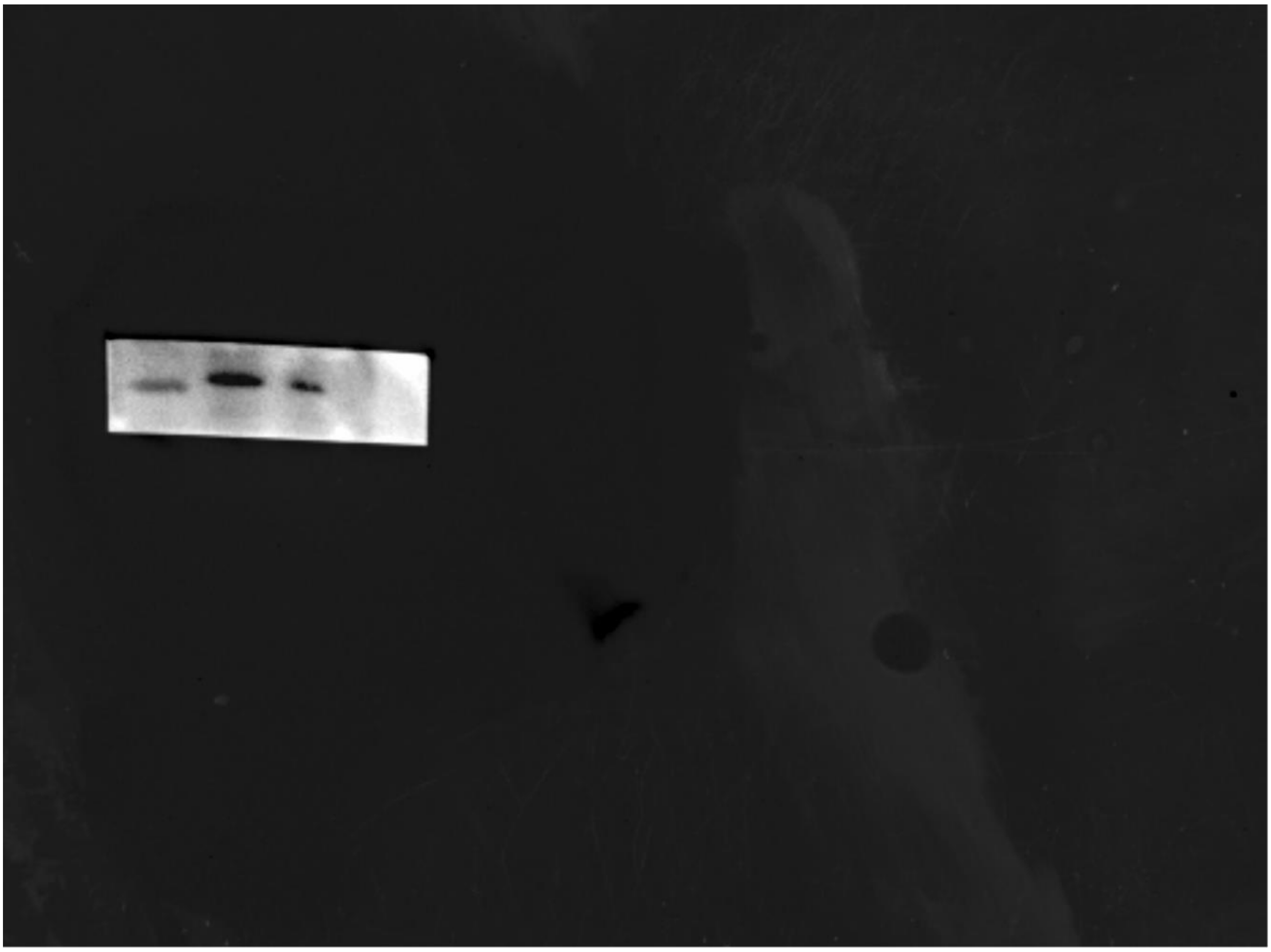

Figure 5A-2

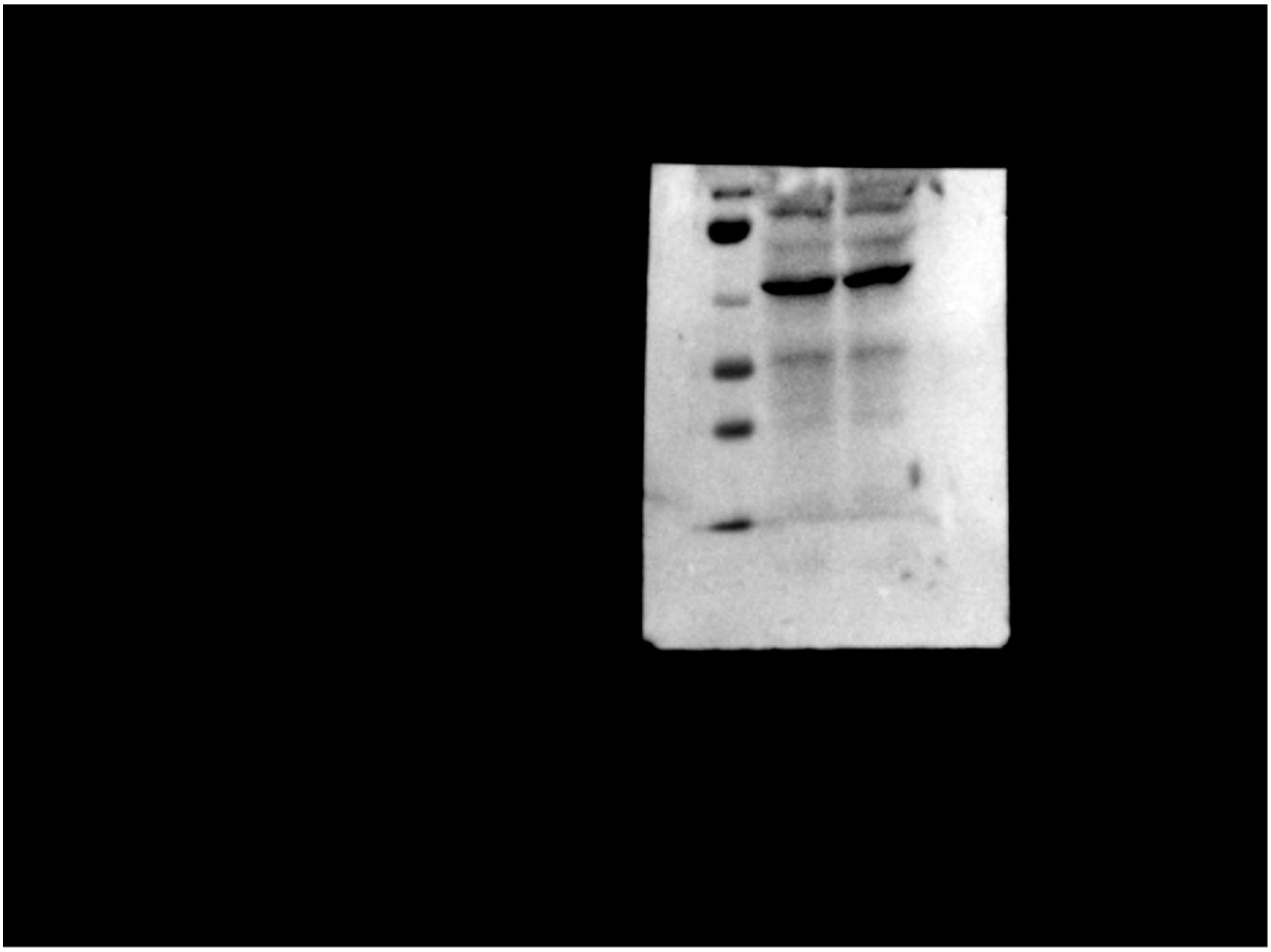

Figure 6A

Figure 6A-1

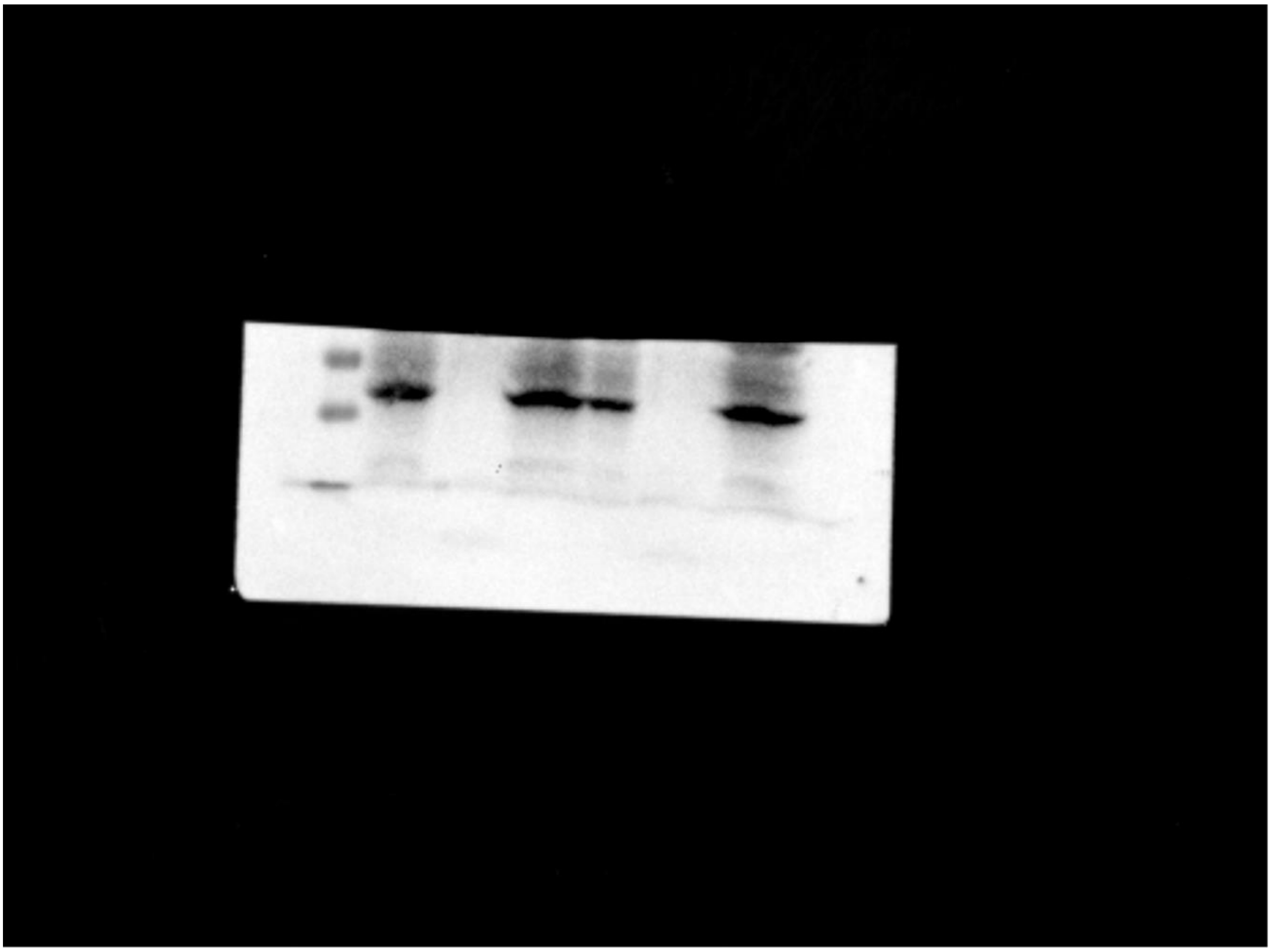

Figure 6A-2

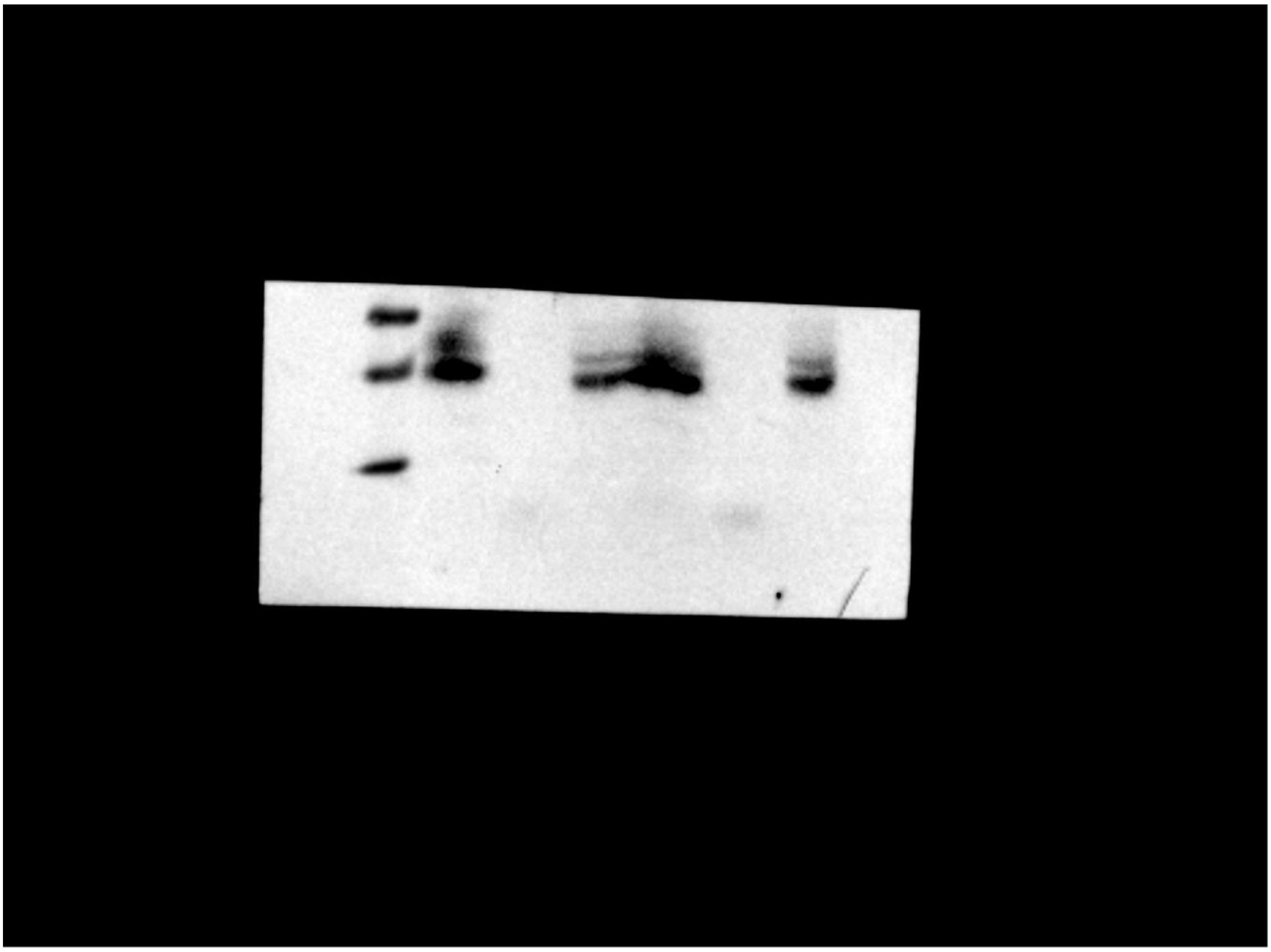

Figure 6A-3

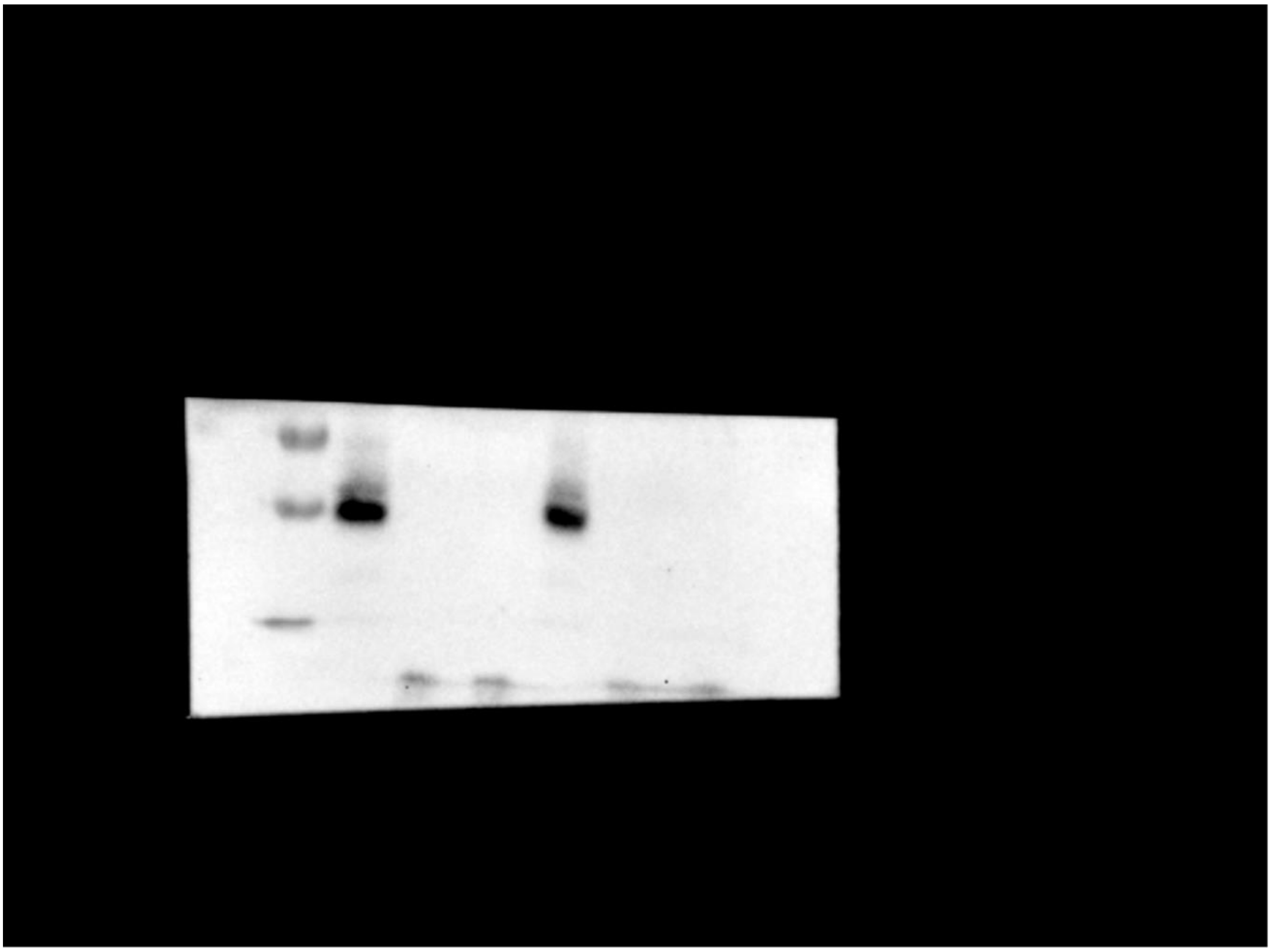

Figure 6B

Figure 6B-1

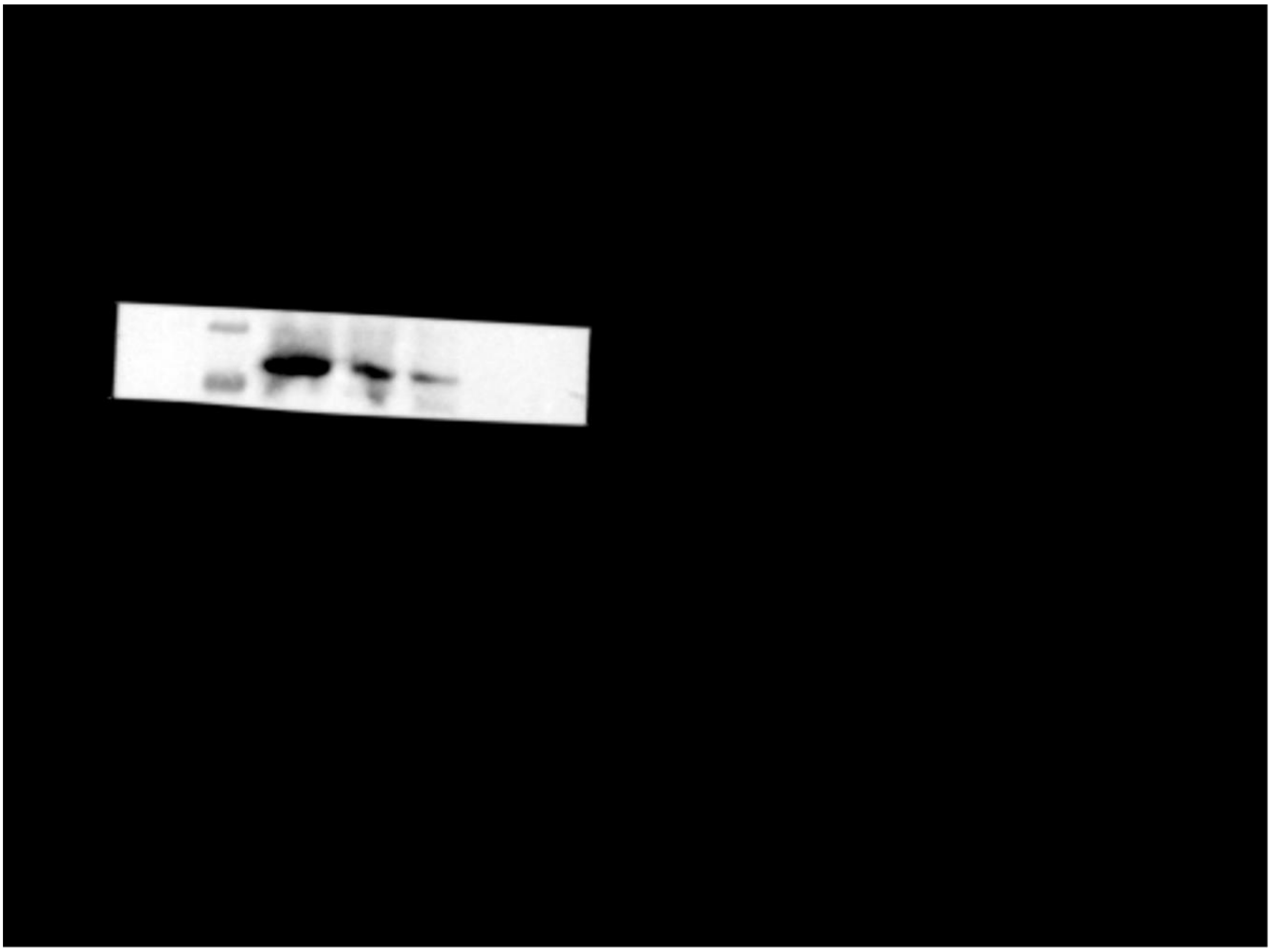

Figure 6B-2

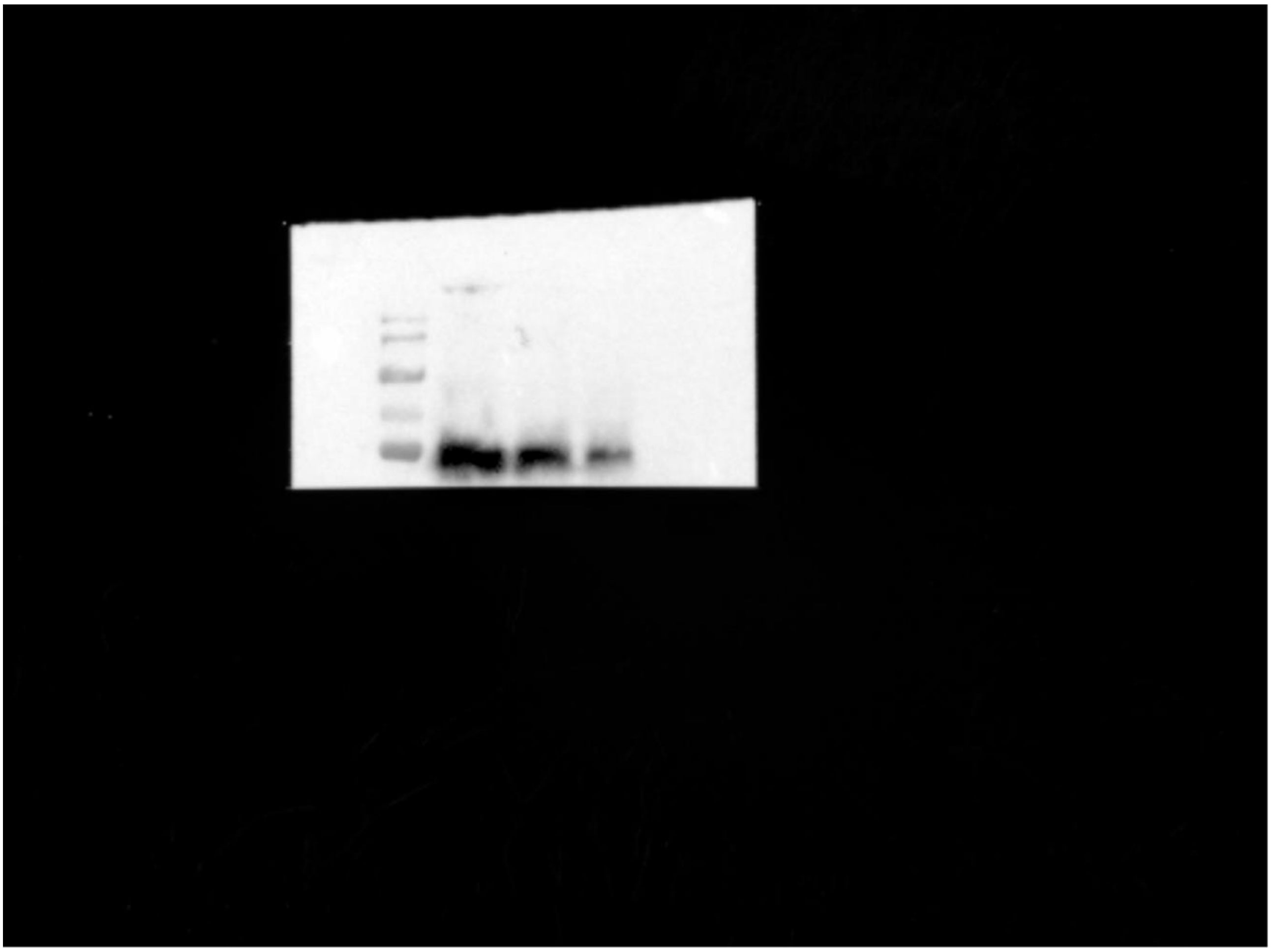

Figure 6B-3

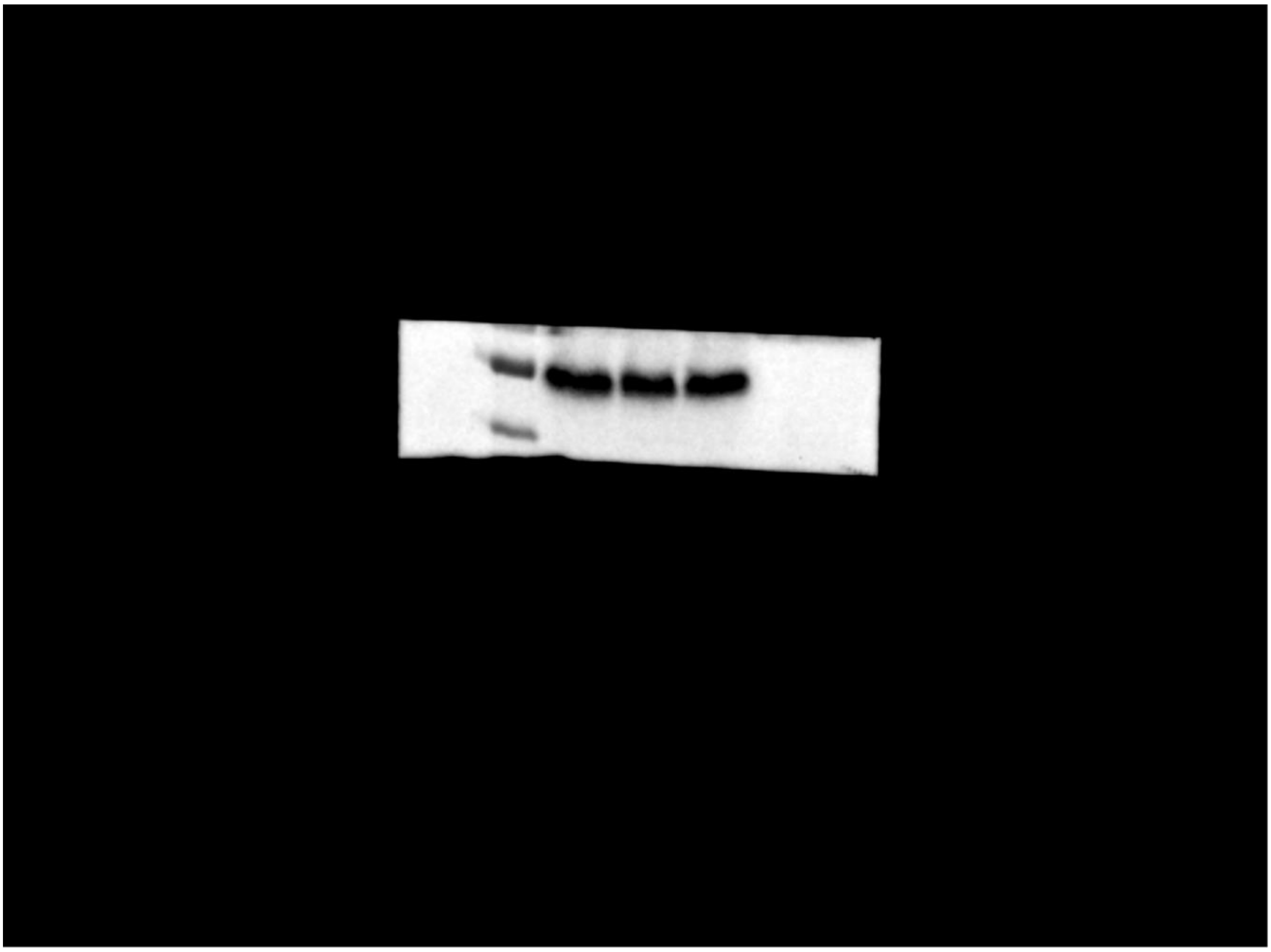

Figure 6C

Figure 6C-1

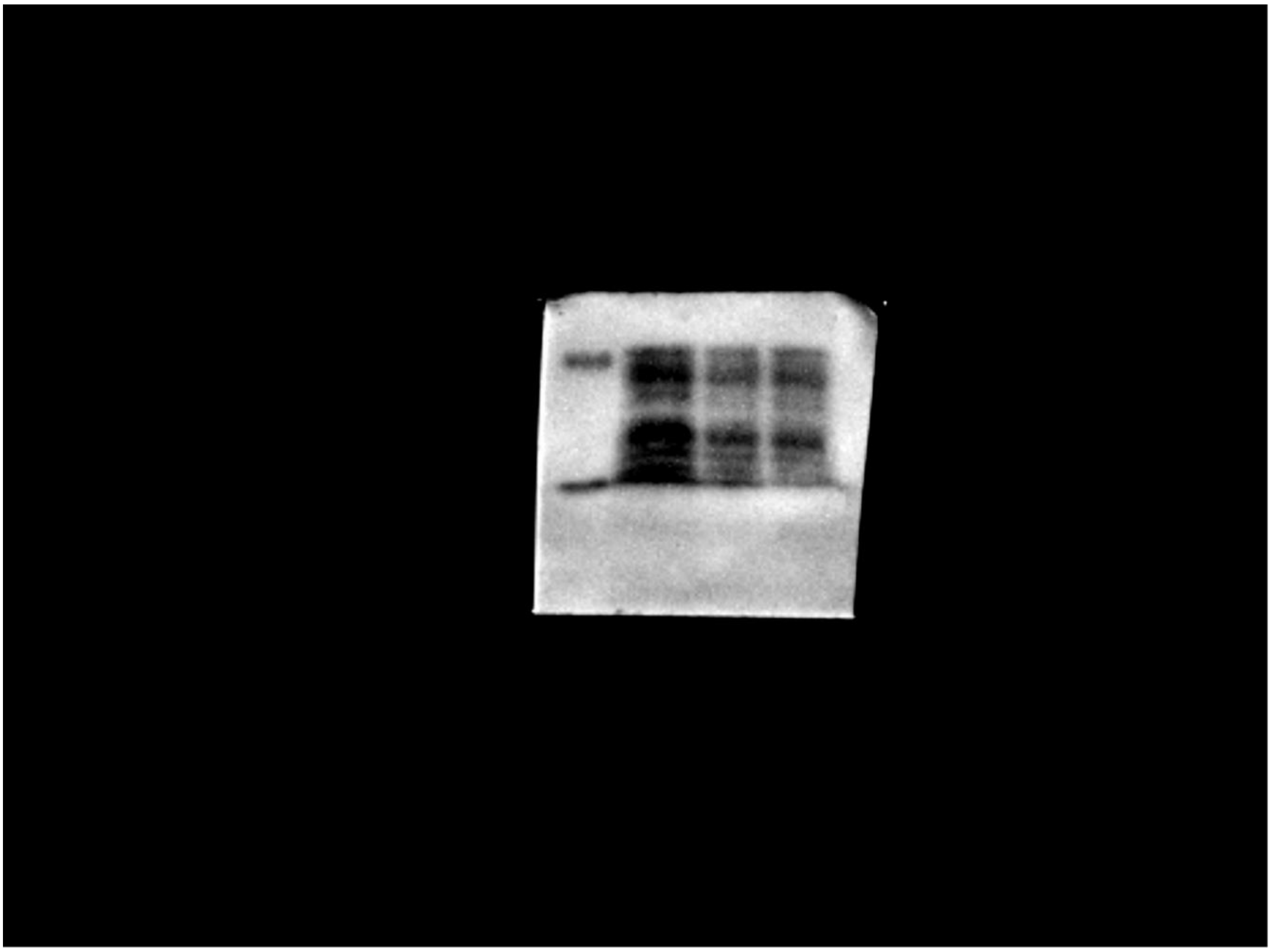

Figure 6C-2

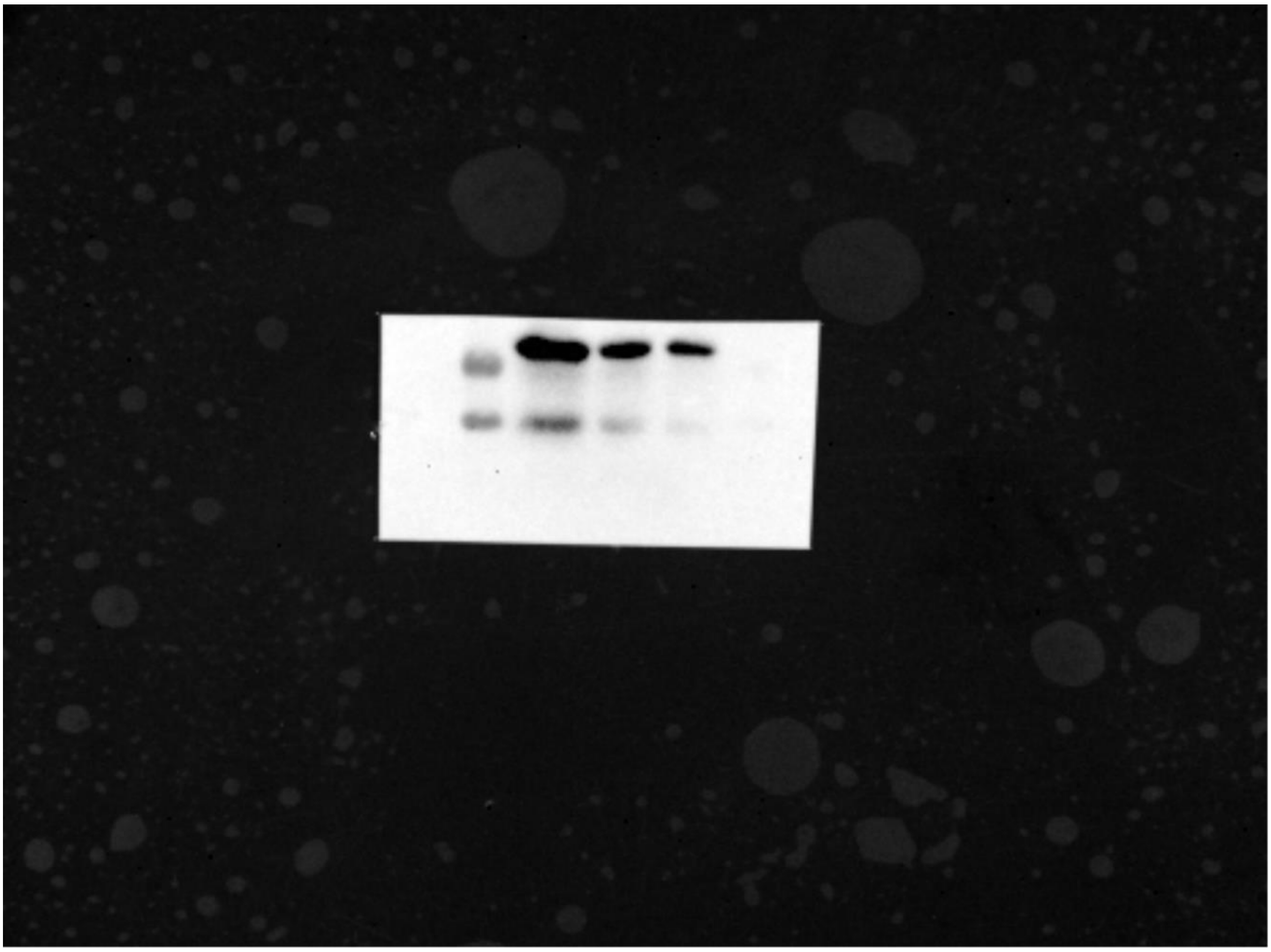

Figure 6C-3

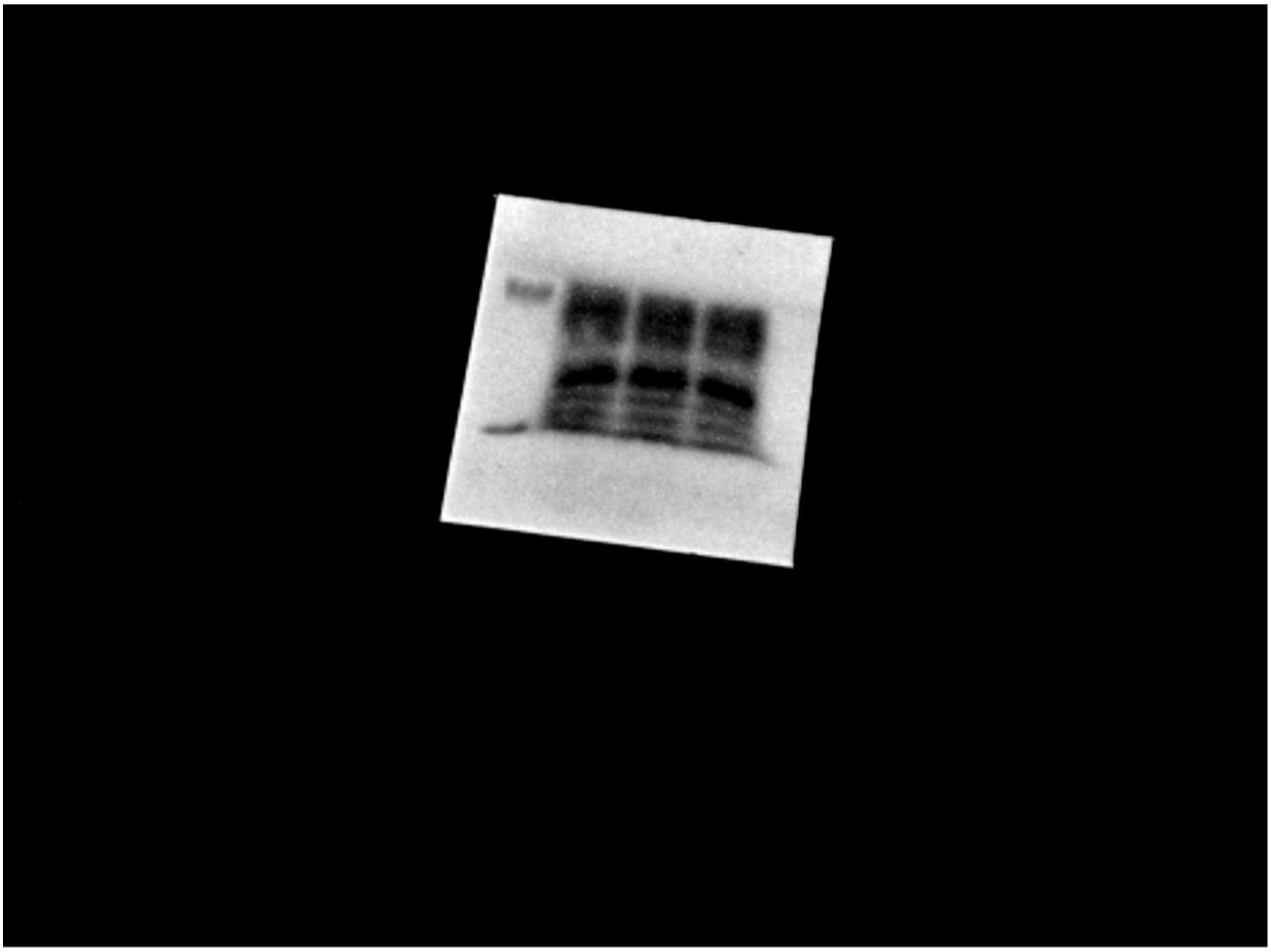

Supplement: Supplementary file 2 — Original Data File [file 41420_2023_1429_MOESM2_ESM.pdf]
